# Combination therapy targeting the tumor microenvironment is effective in a model of human ocular melanoma

**DOI:** 10.1186/1479-5876-5-38

**Published:** 2007-07-18

**Authors:** David P Mangiameli, Joseph A Blansfield, Stephan Kachala, Dominique Lorang, Peter H Schafer, George W Muller, David I Stirling, Steven K Libutti

**Affiliations:** 1 Tumor Angiogenesis Section, Surgery Branch, National Cancer Institute, National Institutes of Health, Bethesda, MD, USA; 2 Celgene Corporation, Summit, NJ, USA

## Abstract

**Background:**

Ocular melanoma is the leading intraocular malignancy. There is no effective treatment for metastatic ocular melanoma. We sought a treatment targeting the tumor microenvironment as well as the tumor cells.

**Methods:**

Migration of HUVEC cells, the ability of HUVEC cells to form tubes, and proliferative capacity of a human ocular melanoma cell line were tested in the presence of lenalidomide and sorafenib alone and in combination. The compounds were also tested in a rat aortic ring assay and were tested in a highly aggressive human ocular melanoma xenograft model.

**Results:**

Lenalidomide and Sorafenib inhibit HUVEC ability to migrate and form tubes and when used in combination the inhibition is increased. The agents alone and in combination inhibit outgrowth in the rat aortic ring model. The combination of the agents improved the inhibition over either single agent. In a xenograft model, combination therapy inhibited tumor growth over inhibition by single agent alone in a significant fashion (p < 0.004: lenalidomide and p < 0.0035: sorafenib). Furthermore, spontaneous lung metastasis development was completely inhibited in the combination treated animals. Sixty percent of vehicle treated animals developed lung metastases compared to 50% of lenalidomide treated animals, and 33% of sorafenib treated animals.

**Conclusion:**

Lenalidomide and sorafenib are effective at targeting endothelial cells, inhibiting growth of ocular melanoma cells and can inhibit growth of tumors in a xenograft model as well as inhibit development of metastases. Combining these agents works in an additive to synergistic way to inhibit the growth of tumors and development of metastases.

## Background

Although ocular melanoma (OM) is a relatively rare diagnosis, it is the leading intraocular malignancy and accounts for approximately 5% of all melanomas. It has a slight male predominance and the age-specific incidence peaks at 70 years of age[1]. Patients can expect a 21–55% chance of developing metastatic disease within 10 years, depending on the size of the primary lesion. For patients who develop metastatic disease, the median survival is < 6 months and virtually all of them will succumb to their cancer. Although OM is known for its metastatic tropism to the liver (89%), it can also be found in a number of other locations, including lung (29%), bone (17%), skin or subcutaneous tissue (12%), lymph node (11%), brain (5%), and other tissues (> 20%)[2]. These sites of disease are not mutually exclusive and they underscore OM's behavior as a systemic disease. Although some regional therapies have modest efficacy in treating hepatic tumor burden, their limitation has remained regional *and *distant recurrence[3,4]. Systemic therapy has unfortunately been ineffective. Aggressive tumor histologies like OM are likely to circumvent cytotoxic therapies, because the only target is cell death. Our approach is to target the tumor microenvironment using agents which have a broad spectrum activity of against the tumor and its vasculature.

Sorafenib (Nexavar™, Bayer), recently approved for the treatment of metastatic renal cell carcinoma, is a bi-aryl urea shown to inhibit multiple receptor tyrosine kinases (RTK) and Ser/Thr kinases[5]. These include but are not limited to: all isoforms of Raf, all isoforms of VEGFR, and PDGFR-β. This multifunctional profile lends itself to inhibition of: tumor and endothelial cell proliferation via the Ras/Raf/MEK pathway, endothelial cell activation and proliferation via VEGFR-2, recruitment of pericytes via PDGFR-β (required for vessel stabilization and maturity), recruitment of stabilizing stromal cells to the tumor's parenchyma, as well as subsequent stimulation of stromal cell derived growth factors [5-10].

Lenalidomide (Revlimid^®^Celgene) is one of the IMiDs^® ^compounds that modulate the immune system and other biologically important targets through multiple mechanisms of action[11]. It is a thalidomide analog approved for the treatment of multiple myeloma and other similar lymphoproliferative diseases. It also has a multifunctional profile and has been shown to cause caspase-dependent apoptosis of tumor cell lines [12,13], inhibit bFGF and tumor induced neovascularization *in vivo *[14,15], abrogate AKT/PKB phosphorylation required for endothelial cell migration[14] and tumor cell proliferation[16],, inhibit proangiogenic TNF-α production[17], and activate and stimulate proliferation of cytotoxic T-cell lymphocytes[18].

The combination of these two compounds addresses the tumor microenvironment as it pertains to tumor cell proliferation and apoptosis, vascular induction and stabilization, immunomodulation, and stromal support. Our hypothesis is that modulation of the tumor's microenvironment with multi-directed therapy, in the form of combinatory treatment with sorafenib and lenalidomide, will have improved efficacy in angiogenesis assays and a human ocular melanoma xenograft model. We therefore studied these agents alone and in combination, in our in vitro, ex vivo and in vivo models. Our goal is to develop more effective agents for the treatment of patients with stage IV OM.

## Methods

### Compound preparation

Sorafenib (Bayer) was obtained from the NIH Pharmacy in the commercially available form of 200 mg tablets. Total pill weight is 350 mg and an index of 1.75 was used to keep the molarity in terms of active ingredient (MW-637 g/mol). Tablets were pulverized with a mortar and pestle and kept from light in a dessicator. Lenalidomide (MW-259.3 g/mol) was obtained from Celgene Corporation. For in vitro and ex vivo studies lenalidomide, sorafenib, oxaliplatin and fumagillin (Sigma-F6771) were solubilized in 100% DMSO on the day of treatment. Oxaliplatin was used as a positive control in these experiments due to its known inhibition of ocular melanoma cells in vitro and fumagillin was used as a positive control secondary to its known inhibition of endothelial cells in vitro. For combinatory treatments, the compounds were kept in 1:1 concentration ratios when solubilized in DMSO, so that the final treatment media kept a final uniformity of 0.1% DMSO. For in vivo studies, sorafenib and/or lenalidomide were suspended in an aqueous solution of 0.5% carboxymethylcellulose and 0.25% Tween 80 (Sigma-C9481 and P8074) immediately prior to cohort gavage.

### Angiogenesis assays

#### Migration assay

The outside undersurfaces of twelve well plates were scribed with a Sharpie permanent marker, so that each well was marked at its largest diameter. Human umbilical vein endothelial cells (HUVEC, ATCC CRL-1730) were then plated in EGM (Cambrex-cc3162) and allowed to reach confluence. After HUVEC reached confluence, EGM was aspirated and cells were washed by submersion and gentle agitation in sterile 37°C PBS without Ca++ and Mg++. A wound in the monolayer was made perpendicular to the scribed line using a P1000 pipette tip. The plate was then washed again by submersion and treatment media was added. Treatment media consisted of EGM-2 (Cambrex-cc3156) with 1% FBS and different concentrations of experimental compound. All groups were kept in a 37°C incubator. Negative control was 0.1% DMSO and when this group reached virtual confluence (~20–24 hours), all plates were fixed with 4% formalin and stained with DAPI. Plates were then imaged with a fluorescent inverted phase contrast microscope (Zeiss). The high power field immediately above and below the scribed line were prospectively designated for analysis, allowing six images per treatment group (Axiovision software). A baseline cohort was fixed, stained and imaged immediately after monolayer wounding. All images were left in their original size format. Quantization was blinded and performed by creating a longitudinal axis over the area of minimal density that corresponded to the site of wound formation. The average baseline wound area was centered over the axis and all cells that were present with in that area were assumed to have migrated there. These cells were counted and the cell counts constituted raw data for analysis.

#### Tube formation assay

Matrigel (BD Biosciences-354234) was plated at 200 ul/well in 24 well plates and allowed to reach the solid phase after one hour in a 37°C incubator. HUVEC were then suspended in treatment media identical to the migration assay and plated on top of the Matrigel at a density of 50, 000 cells per well. After six hours in an incubator, the wells were imaged on an inverted phase contrast microscope (Zeiss, Axiovision). These images were used to derive data. HUVEC normally form a branching plexus of tubes on artificial extracellular matrices, such as Matrigel. Quantization was blinded and performed by counting each nodal branch point that had 3 or more branches. Branch point counts per image constituted the raw data for statistical analysis. There were four images per treatment group.

#### Rat aortic ring assay

Matrigel was plated at 250 ul/chamber on CultureSlides (BD 354104) and allowed to solidify overnight at 37°C. Next, six week old Sprague-Dawley rats were sacrificed and their thoracoabdominal aortas procured. The aortas were dissected free of any fibro-adipose tissue and sectioned into 0.5 mm rings. The rings were kept in EGM-2 media in a 50 ml conical tube while other animals (total of four) were being processed. Gentle agitation of the conical tube constituted randomization of any one animal's contribution to a particular treatment group. All rings that had branches or were eccentric were removed. Rings were then placed on the Matrigel layer, one ring per chamber. Each ring was then embedded in Matrigel by the addition of another 200 ul. These were allowed to incubate for one hour at 37°C and EGM-2 media was added. The rings were incubated for another 24 hours and the next day media was exchanged for basal media containing various concentrations of drug and 0.1% DMSO. The rings were incubated in treatment media for five days, with media and drug compound being refreshed every other day, after five days they were imaged on an inverted phase contrast microscope (Zeiss). All images were acquired (Axiovision) via the same settings and in the same sitting. The images were imported into Adobe Photoshop CS2 and were not modified in size, shape or contrast, in any way. Blinded quantization was done using Photoshop's magic wand function to select the pixel densities associated with the vascular sprouts of each ring. Cutting and pasting allowed confirmation that all and only sprouts were selected; the aortic ring itself was excluded. Photoshop's expanded histogram was then used to yield pixel counts that represented the selection. These pixel counts served as raw data for analysis.

### Real-time cell electronic sensing

92.1 cells were grown to subconfluence in RPMI 1640 with 10% FBS. The monolayer was trypsinized, counted (trypan blue exclusion), and resuspended in complete media at a density of 1 × 10^5 ^cells/ml. The ACEA RT-CES 16× E-Plate Station (ACEA Biosciences, San Diego, CA) was used in an incubator at 37°C and 5% CO_2_. 100 ul of complete RPMI 1640 was added to the wells of 16× E-Plates (ACEA Biosciences) after which they were allowed to acclimate in the incubator for 30 minutes. ACEA RT-CES SP software (ACEA Biosciences) was then used to calibrate the plates. 100 ul of cell suspension was added to the plates and the next day treatment with compound in DMSO was added to the wells, so that the final concentration of DMSO was uniformly 0.1%.

### Human ocular melanoma xenograft model

All animal studies were in accord with the National Institutes of Health-Animal Care and Use Committee guidelines. Female NCr-*nu/nu *mice (Taconic Farms, NCI, Animal Production Program, Frederick, Maryland) were used for all tumor challenge experiments. The ocular melanoma cell line 92.1 (gifted by Bruce R. Ksander, Harvard Medical School) was maintained in culture using RPMI-1640 with 10% FBS. Cells were trypsinized while in their log growth phase, resuspended in 50% Matrigel and 50% complete RPMI, to a volume that provided a final cell density of 1 × 10^8 ^cells/ml. Mice were then dorsally injected subcutaneously with 100 ul of cell suspension, using a 27 g needle. When lesions showed evidence of progression (~10–14 d), they were randomized to treatment groups. Therapy involved daily oral gavage of 100 mg/kg lenalidomide and/or 60 mg/kg sorafenib in 50 ul doses through a 22 g oral gavage needle. Subcutaneous lesions were measured in three dimensions on a MWF schedule. Tumor volumes [0.52(L × W × H)] constituted the raw data for analysis. After 14 days of treatment, animals were euthanized and their subcutaneous lesions were resected and immediately snap frozen in liquid nitrogen. In order to count visceral surface lung metastases, the tracheobronchopulmonary tree was resected en bloc and the trachea was cannulated with a 21 g needle and insufflated with 10% formalin. The specimens were then stored in formalin overnight and the next day, the counts were blindly performed with the use of 2× surgical loupes and a dissecting microscope.

### Statistical method

Statistical analysis was performed with the use of GraphPad InStat v.3.05, GraphPad Prism v.4.02, and Excel 2002. Statistical analysis of the migration and tube formation assay data involved One-way ANOVA followed by Tukey-Kramer multiple comparisons testing. The rat aortic ring data underwent One-way ANOVA followed by Tukey-Kramer multiple comparisons testing if assumption testing by Bartlett's method revealed no differences in the standard deviations between groups. If there was a disparity in inter-group standard deviations, then the nonparametric Kruskal-Wallis test was used, followed by Dunn's multiple comparisons method. Relationships of significance were then applied to unpaired Student's T test with Bonferroni adjustment for final p values. Data from the xenograft model were evaluated with One-way ANOVA with multiple comparisons testing. This also was then applied to unpaired Student's T test with Bonferroni adjustment for final p values. The xenograft outcomes analysis was made on the basis of given measurement days.

## Results

### Lenalidomide

#### Migration assay

**Figure 1:**
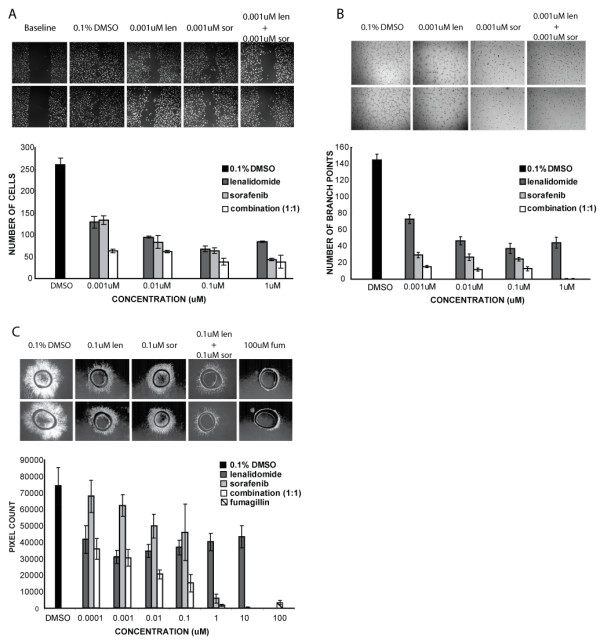
(A) Migration assay data for single agent lenalidomide, sorafenib and combination treated HUVEC reveals a dose response curve that has significant inhibition at all concentrations. Treatment of HUVEC with a 1:1 combination of lenalidomide and sorafenib showed superior inhibitive efficacy than monotherapy with either compound. There was no detectable difference between lenalidomide or sorafenib monotherapy for the migration assay. Each experimental condition was repeated six times. (B) Tube formation assay showed inhibition of HUVEC ability to form tubes with a maximal inhibition with lenalidomide treatment at 0.001 uM (p = 0.005). The tube formation capabilities of HUVEC were more profoundly inhibited by sorafenib than lenalidomide at all tested concentrations. The combination of compounds showed superior inhibitory efficacy at all concentrations. Each experimental condition was repeated four times. (C) Lenalidomide inhibited the rat aortic ring assay more effectively at lower concentrations than did sorafenib, and its inhibition was present at across all concentrations. Combination treatment with both compounds was more effective at all evaluable concentrations. Images are representative of those used for data derivation. Each experimental condition was repeated eight times.

Endothelial cells in monolayer normally migrate toward regions of lower population density. Migration, together with proliferation lends itself to uniformity of density. We used the migration assay with HUVEC, to see if endothelial cells sustained a functional deficit in motility when subject to lenalidomide. By making a wound in a confluent monolayer of HUVEC and subjecting the cells to basal treatment media, we minimized the level of proliferation and were able to blindly quantitate the cellular migration capacities and how they were affected by our treatments (Fig. 1A). Inhibition was statistically evident at all tested concentrations of lenalidomide. The 0.01 μM group exhibited the maximum inhibition (67%), p < 0.002.

#### Tube formation assay

Human endothelial cells normally form tubes and branching networks when cultured in the presence of a three dimensional supportive matrix. We evaluated whether their ability to fulfill this role was affected by the presence of lenalidomide, by resuspending HUVEC in treatment media and plating them on Matrigel. HUVEC's ability to form branching tubes was significantly reduced (Fig. 1B). The maximum inhibition occurred at 0.001 μM (73%) p < 0.0005.

#### Human ocular melanoma xenograft model

**Figure 2:**
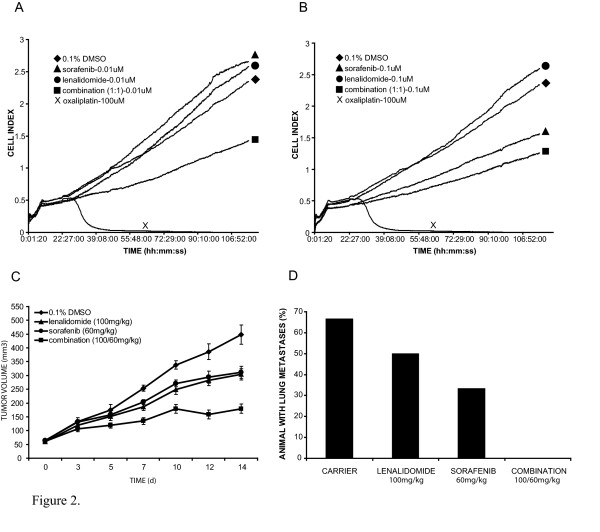
Evaluation of 92.1's proliferative potential was done with RT-CES (A and B). Each experimental condition was repeated eight times. This technology yielded kinetic growth data which showed combination treatment with lenalidomide and sorafenib (1:1) to have an anti-proliferative effect, which is first apparent at 0.001 μM and most profoundly independent from either single agent's effect at 0.01 μM (A). Lenalidomide alone showed no appreciable activity against proliferation regardless of concentration. Sorafenib alone exhibited anti-proliferative activity which was initially evident at 0.1 μM (B), 10–100× more concentrate than when a similar effect is produced by combination therapy. When therapy was evaluated with the xenograft model, the combination treatment cohort showed the most tumor growth delay Each treatment group represents eight animals. (C). Lenalidomide and sorafenib both displayed tumor growth stasis which was significantly different from carrier treated animals and equivocal from each other. Combinatory therapy showed significant growth retardation relative to either monotherapy. This pattern of anti-tumor efficacy was also seen in the analysis of metastatic frequency. Each treatment group represents eight animals (D).

Our ultimate goal is to evaluate compounds for the treatment of patients with ocular melanoma. In order to do this, we developed a xenograft model from a human ocular melanoma cell line, and evaluated lenalidomide's ability to mitigate tumor growth and lung metastases. The primary endpoints were mean tumor volume and presence or absence of visceral surface lung metastases after a fourteen day treatment regimen (Fig 2C and 2D). Lenalidomide treated mice exhibited delayed tumor growth (p < 0.0001), which by day fourteen exhibited a 52% relative reduction in mean tumor volumes. After sacrifice of the animals the lungs were blindly evaluated for number of visceral surface metastases. All animals developed lung metastases of which the control group developed a median of 26.5 lung lesions per animal (CI = 17.57–33.76) and the treatment group developed a median of 12 lung lesions per animal (CI = 10.38–16.20, p = 0.0047).

### Lenalidomide and sorafenib

#### Anti-vascular assays

Results of the migration assay showed that inhibition of migration was statistically evident for all tested concentrations of all treatments (Fig. 1A). Sorafenib and lenalidomide were equivalent, except at the 1 μM concentration, where sorafenib displayed more anti-migration activity (p = 0.0015). In the 0.001 μM cohort, the combinatory arm showed greater inhibition of migration than lenalidomide (p < 0.05) or sorafenib (p < 0.01) alone. Tube formation and branching capabilities were also stunted by all treatment groups at all concentrations (Fig. 1B). Although sorafenib exhibits more inhibition than lenalidomide, combinatory treatment was more inhibitive than either single treatment group at the 0.001 μM (p < 0.05) and 0.01 μM (p < 0.05) concentrations. We then sought to test whether the compounds had any effect on the development of neovasculature from a mature preexisting artery. To test this we used the rat aortic ring assay (Fig. 1C). Lenalidomide showed significant inhibition at all concentrations tested and sorafenib showed significant inhibition at and above the 0.01 μM concentration; lenalidomide was statistically more effective at lower doses. The combinatory treatments were significantly effective at inhibiting neovascular outgrowth at all concentrations, and were significantly more inhibitive than treatment with lenalidomide alone at the 0.01 μM (p = 0.001) and 0.1 μM (p = 0.005) and sorafenib alone at the 0.01 μM (p = 0.0005) and 0.1 μM (p = 0.0015) doses.

#### Anti-tumor assays

##### Real-time cell electronic sensing

After noting the anti-vascular activity of combinatory treatment and given the known biologic targets of lenalidomide and sorafenib, we evaluated whether there was a similar effect on the proliferative potential of a human ocular melanoma cell line. The 92.1 cell line was used in order to make correlations with xenograft data. RT-CES of the 92.1 cell line provided a reproducible dynamic evaluation of cell population growth curves. Analysis of this data revealed growth kinetics of 92.1 in the presence of different compounds at different concentrations (Fig. 2A and 2B). Lenalidomide displayed no appreciable effect on cell index (a dimensionless unit which is proportional to the actual cell number and is a surrogate index of net proliferation and apoptosis[19]). Sorafenib exhibits an anti-proliferative dose response, which first becomes evident at 0.1 μM and becomes more profound with increasing concentration. Combination (1:1) treatment of 92.1 cells resulted in a dose response that is initially evident at 0.001 μM, but is most profoundly differential from individual monotherapies at 0.01 μM. By adding lenalidomide, in the form of combination treatment, the sorafenib dose response is transposed to the left by one to two orders of magnitude concentration.

##### Xenograft

After we found potentiated anti-vascular activity, as well as anti-proliferative activity in the tumor cell line, we wanted to investigate whether there was an increase in anti-tumor activity in vivo, relative to monotherapy. In order to do this, we used our human ocular melanoma xenograft model with the priory endpoint of mean tumor volume after fourteen days of treatment (Fig. 2C). Secondarily, we looked at time to significant tumor growth delay and frequency of lung metastasis (Fig. 2D). The combination treatment group showed improved tumor growth stasis relative to lenalidomide (p = 0.004) and sorafenib (p = 0.0035) at day 14. Inhibition of tumor growth relative to carrier treated animals, was statistically evident by day 7 for lenalidomide (p = 0.0185), sorafenib (p = 0.027) and combination (p = 0.0005) treatment cohorts. Combination therapy was associated with significantly lower mean tumor volumes than sorafenib by day 7 (p = 0.005) and lenalidomide by day 12 (p = 0.003). Visceral pulmonary surface metastases were evident in 66% of carrier treated, 50% of lenalidomide treated, 33% of sorafenib treated, and 0% of combination therapy treated animals. Mice were followed for weight gain or loss, body temperature changes, skin changes as well as for behavioral changes such as restlessness or aggression. There was no evidence of any treatment related toxicity in mice treated with lenalidomide alone, sorafenib alone or the combination of the two agents.

## Discussion

Historically, the efficacy of chemotherapy has been based on how well it could inhibit the growth of tumor cells. More recently, the tumor microenvironment has been shown to play a vital role in the spread of a primary tumor as well as a tumor's ability to metastasize. The tumor microenvironment is made up of a complex network of tumor, endothelial, lymphatic and fibroblast cells on an extracellular matrix. These cells all have a role in the growth of the tumor and as such agents must target multiple aspects of the microenvironment to be effective.

Our lab has tested two agents which target different pathways and different cell types in the tumor microenvironment. We have shown that lenalidomide is able to inhibit the function of endothelial cells in in vitro assays. Lenalidomide inhibits endothelial cells from creating tubes and also inhibits endothelial cells from migrating but does not have a direct cytotoxic effect on the endothelial cells. Furthermore, in an ex vivo assay testing how the compound affects the entire tumor microenvironment, lenalidomide inhibits the outgrowth of buds from the rat aortic ring. These observations of inhibition of endothelial tubule formation and migration, as well as inhibition of microvessel sprouting in the rat aortic ring model, along with a lack of an effect on endothelial cell proliferation, are consistent with previous reports on the activity of lenalidomide. [14,15] In our experience, the rat aortic ring is one of the best predictors of how an agent will affect tumors in vivo because it functions as a microcosm of the tumor microenvironment. In a highly aggressive in vivo model of a human ocular melanoma, lenalidomide alone was able to slow the growth of subcutaneous tumors grown in the mouse, as well as reduce the number of lung metastases.

Although lenalidomide had an anti-angiogenic effect in several in vitro, and ex vivo assays, and an effect on in vivo tumors, we wanted to improve on the effect by adding a compound to target additional pathways which lenalidomide might not inhibit and thus disrupt more pathways in the complex tumor microenvironment. In addition, the combination may decrease the ability of the tumor to escape the effects of a single agent by compensatory mechanisms we chose sorafenib, based on its ability to block several of the receptors responsible for endothelial cell growth and function: namely VEGFR, and PDGFR-beta. Furthermore, sorafenib is able to block the Ras kinase pathway which has been shown to be active in melanoma tumor cells. This combination of a Ras/MAPK pathway inhibitor in combination with lenalidomide, which has been shown to interfere with AKT signaling in endothelial cells[14] and tumor cells[16], is perhaps advantageous because it simultaneously blocks signaling through both the MAPK and AKT pathways. As suspected, the addition of sorafenib improved the inhibition of endothelial cell function in vitro as well as enhanced the inhibition of cells which make up the microenvironment as shown in the rat aortic ring model. Sorafenib was also shown to have a direct cytotoxic effect on our human ocular melanoma cell line in an assay of cell proliferation. The agents were able to slow the growth of tumors without causing toxicity to the mice and with combination treatment there was no added toxicity. Most importantly, in a highly aggressive in vivo model of human ocular melanoma, the combination of lenalidomide and sorafenib was able to inhibit the growth of subcutaneous tumors as well as inhibit the growth of metastatic deposits in the lungs more effectively than shown by either compound itself.

## Conclusion

Lenalidomide is an IMiD which can inhibit the function of endothelial cells in vitro, can block the outgrowth of cells from a rat aortic ring mimicking the inhibition of cells in the tumor microenvironment, and can inhibit the growth of a primary tumor as well as inhibit the growth of metastases in a human ocular melanoma xenograft model. Sorafenib likewise can inhibit endothelial cells in vitro, ex vivo and can inhibit tumor growth in vivo. Further, the combination of lenalidomide and a tyrosine kinase inhibitor like sorafenib is a viable combination which targets multiple aspects of the tumor microenvironment in vitro, ex vivo and in vivo. Based on our pre-clinical results, we believe that this combination and this strategy warrant testing in a clinical setting against ocular melanoma.

## Abbreviations

HUVEC: human umbilical vein endothelial cells

OM: ocular melanoma

bFGF: basic fibroblast growth factor

TNF-alpha: tumor necrosis factor- alpha

IMiD: immunomodulatory drug

RTK: receptor tyrosine kinase

VEGF and VEGFR: vascular endothelial growth factor (receptor)

PDGF and PDGFR: platelet derived growth factor (receptor)

## Competing interests

The author(s) declare that they have no competing interests.

## Authors' contributions

DM participated in the design of the study, carried of the in vitro, ex vivo studies, and in vivo studies, drafted the manuscript and performed the statistical analysis. JB participated in the design of the study, carried of the in vitro, ex vivo studies, and in vivo studies, drafted the manuscript and performed the statistical analysis. SK and DL participated in the design and coordination of the study and helped carry out the in vitro and in vivo studies. PS, GM, and DS participated in the design of the study and helped draft the manuscript. SKL helped to conceive the study, participated in its design and coordination, oversaw the implementation of the study and helped draft the manuscript. All authors read and approved the final manuscript.
